# Effects of the Incorporation of Distinct Cations in Titanate Nanotubes on the Catalytic Activity in NO_x_ Conversion

**DOI:** 10.3390/ma14092181

**Published:** 2021-04-24

**Authors:** José Vitor C. do Carmo, Cleanio L. Lima, Gabriela Mota, Ariane M. S. Santos, Ludyane N. Costa, Anupama Ghosh, Bartolomeu C. Viana, Monique Silva, João M. Soares, Samuel Tehuacanero-Cuapa, Rossano Lang, Alcineia C. Oliveira, Enrique Rodríguez-Castellón, Elena Rodríguez-Aguado

**Affiliations:** 1 Department of Analytical and Chemical-Physic Chemistry, Pici Campus-Block 940, Federal University of Ceará, Fortaleza 60040-531, Brazil; vitor.costa@alu.ufc.br (J.V.C.d.C.); gabrielamotab@alu.ufc.br (G.M.); 2 Material Science and Engineering & Physics Department, Federal University of Piauí, Teresina 64049-550, Brazil; cleanio@ufpi.edu.br (C.L.L.); arianeo_1q@bol.com.br (A.M.S.S.); luydiane@bol.com.br (L.N.C.); anupama1984@gmail.com (A.G.); bartolomeu@ufpi.edu.br (B.C.V.); 3 Fortaleza Campus, Federal Institute of Education—IFCE, Av. 13 de Maio, 2081, Benfica, Fortaleza 60040-531, Brazil; moniquessouza22@gmail.com; 4 Physics Department, State University of Rio Grande do Norte-UERN, BR 110-km 48, R. Prof. Antônio Campos, Costa e Silva, Mossoró 59610-210, Brazil; joaomsoares@gmail.com; 5 Central Microscopy Laboratory, Physics Institute—UNAM, Research Circuit s/n, University City, Coyoacán, Mexico City 04510, Mexico; samueltc@fisica.unam.mx; 6 Institute of Science and Technology—ICT, Federal University of São Paulo—UNIFESP, São José dos Campos 12231-280, Brazil; rossano.lang@unifesp.br; 7 Department of Inorganic Chemistry, Faculty of Science, University of Málaga, 29071 Málaga, Spain; aguadoelena5@gmail.com

**Keywords:** PtO_x_ species, impregnation, nanoparticles, titanate nanotubes, NO_x_

## Abstract

Effects of the incorporation of Cr, Ni, Co, Ag, Al, Ni and Pt cations in titanate nanotubes (NTs) were examined on the NO_x_ conversion. The structural and morphological characterizations evidenced that the ion-exchange reaction of Cr, Co, Ni and Al ions with the NTs produced catalysts with metals included in the interlayer regions of the trititanate NTs whereas an assembly of Ag and Pt nanoparticles were either on the nanotubes surface or inner diameters through an impregnation process. Understanding the role of the different metal cations intercalated or supported on the nanotubes, the optimal selective catalytic reduction of NO_x_ by CO reaction (SCR) conditions was investigated by carrying out variations in the reaction temperature, SO_2_ and H_2_O poisoning and long-term stability runs. Pt nanoparticles on the NTs exhibited superior activity compared to the Cr, Co and Al intercalated in the nanotubes and even to the Ag and Ni counterparts. Resistance against SO_2_ poisoning was low on NiNT due to the trititanate phase transformation into TiO_2_ and also to sulfur deposits on Ni sites. However, the interaction between Pt^2+^ from PtO_x_ and Ti^4+^ in the NTs favored the adsorption of both NO_x_ and CO enhancing the catalytic performance.

## 1. Introduction

Titanate nanotubes are fascinating solids that have spurred significant interest as catalysts for several reactions [1,2]. These features include the metal/metal oxide intercalation or decoration that exists in titanate nanotubes, allowing their application in catalytic reactions [1,2,3,4,5].

Among these catalytic reactions, selective catalytic reduction (SCR) is likely to be the most effective technology for reducing nitrogen oxides, e.g., NO_x_ emissions, owing to its simpler and continuous operation, greater flexibility for the use of reducing agents, lower costs and eco-friendliness requirements [5,6,7,8,9,10,11] (and references herewith). SCR using NH_3_ (NH_3_-SCR) as a reducing agent is especially effective in converting NO_x_ along with its distinctive way of treating exhaust gas from stationary sources and power plants [5,8,9,10].

Nonetheless, the NH_3_-SCR process is still constrained by several challenges, particularly the low resistance to SO_2_ and H_2_O poisoning and the formation of ammonium sulfates deposit on the surface of the commercial V_2_O_5_-WO_3_(MoO_3_)/TiO_2_-based catalyst [5,6,7]. This results in the deactivation of the catalysts due to the competitive adsorption of the reactants and SO_2_, formation of metal and ammonium sulfates, water vapor inhibition of the acid sites, as well as production of undesired NO and N_2_O by-products from NH_3_-SCR reaction instead of nitrogen, the desired compound [8,9].

**Figure A1:**
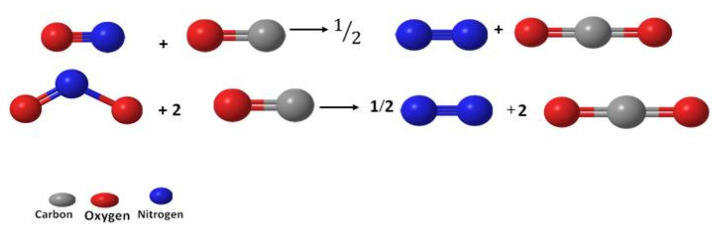
Schematic representation of the reactions that occur during CO-SCR reaction.

Numerous researchers have attempted to improve SCR processes by optimizing the physicochemical properties of the catalysts and changing reducing agents. Nowadays, substantial interest has been devoted to the SCR using hydrocarbons (HC-SCR), e.g., CH_4_, C_3_H_6_, C_4_H_10_ among others as the primary reductants for controlling NO_x_ emissions over a variety of catalysts [12,13,14]. In this context, CO-SCR is a very versatile reaction between nitrogen oxide and carbon monoxide, whereby the reactants can be further transformed into nitrogen and carbon dioxide (Figure A1, Appendix A).

Supported transition metal catalysts, particularly those possessing dispersed active sites on titania, alumina, silica, ceria, molybdena, zirconia, molecular sieves, zeolites and lantana have long been used as catalysts for the CO-SCR reaction [6,13,14,15,16] (and references herewith). However, the use of these catalysts in the presence of SO_2_ and water vapor poisons, especially in low-temperature regimes, results in a considerable loss of activity and deactivation of catalysts, besides the complicated poisoning of the active sites by SO_2_ to form transition stable and inert metal sulphates on the surface of the catalysts [8,14,15,16,17,18,19].

Currently, Cu, Pt, Ni, Mn, Pd, Rh, Ag oxides have been commonly used as active components of the catalysts for the decomposition of NO_x_ through SCR reactions. Moreover, it is shown that the introduction of promoters into the catalysts and the removal of reaction inhibitors during the SCR avoiding side reactions enhance the catalytic performance [6,8] (and references herewith).

Amongst these catalysts, metal-containing titanate nanotubes have shown significant enhancement in the catalytic efficiency for decomposition of NO_x_ reactions, regarding selectivity and yield to N_2_ and carbon monoxide products ([8,9,10,20,21,22,23,24] and references herewith). With the use of metal center intercalated into the titanate nanotubes structure, the NH_3_-SCR reaction proceeds in the presence of a bifunctional catalyst through the formation of intermediate species such as hydrogen cyanide (HCN), isocyanate (–NCO), nitromethane (CH_3_NO_2_) and oxygenated hydrocarbons (C_x_H_y_O_z_) [5,8,18], which are accompanied by nitrogen and carbon dioxide.

Although these solids have gathered much information, they did not show details about the catalyst’s active site features, neither it was proposed proper reaction conditions to apply the solids. Moreover, the mechanistic consideration of the role of the metal sites remains under debate. Therefore, there is still interest in developing titanate nanotubes-based catalysts for the CO-SCR to define the role of the different metal sites in the catalytic performance and gain insights into the best parameters for this reaction.

However, to the best of our knowledge, no reports are given on the use of metal-containing titanate nanotubes for the CO-SCR reaction.

In this paper, for the first time, the properties of titanate nanotubes were systematically studied by introducing different transition-metal ions and evaluated in the CO-SCR reaction. Moreover, the tubular morphology of NTs offers the advantages of the nanotubes acting as nanoreactors for chemisorption of the reactants during NO_x_ conversion through CO-SCR reaction, as compared to bulk TiO_2_ and Ti-based nanoparticles counterparts.

The effects of the metal intercalated into the titanate nanotubes and their dispersions and tolerance to poisons on the catalytic performances are investigated during CO-SCR reaction.

## 2. Experimental Part

### 2.1. Materials

TiO_2_ anatase powder, chromium(III) nitrate nonahydrate (Cr(NO_3_)_3_·9H_2_O), cobalt(III) nitrate (Co(NO_3_)_3_), nickel(II) nitrate hexahydrate (Ni(NO_3_)_2_·6H_2_O), aluminum(III) nitrate nonahydrate (Al(NO_3_)_3_·9H_2_O), silver(I) nitrate (AgNO_3_) salts and aqueous sodium hydroxide solution (NaOH (aq), 10 mol·L^−1^) were purchased from Vetec (Vetec, São Paulo, Brazil). The hexachloroplatinic acid solution (H_2_PtCl_6_·6H_2_O solution 8 wt% in water) was purchased from Sigma-Aldrich (Sigma-Aldrich, St. Louis, MO, USA). All reactants were used as received.

### 2.2. Synthesis of the Catalysts

Titanate nanotubes (NTs) were synthesized by a hydrothermal method [2,25]. Typically, a solution containing 10 mol·L^−1^ of sodium hydroxide was added to two grams of TiO_2_ anatase (25 mmol) with constant stirring. Then, the mixture was placed in a Teflon container (WT Indústria, São Carlos, São Paulo, Brazil) at 160 °C for 72 h in an autoclave (WT Indústria, São Carlos, São Paulo, Brazil). The solid was afterwards separated by vacuum filtration (Vacuum desiccator, Laborglas, São Paulo, Brazil) and copiously washed with deionized water to remove the excess amount of hydroxyl ions. Finally, the catalyst was dried at 50 °C overnight to obtain the sodium-containing trititanate nanotubes powder (NaNT).

Metal-containing titanate nanotubes (MeNT) were prepared by ion-exchange using aqueous solutions of chromium(III), cobalt(III), nickel(II), silver(I) and aluminum nitrate salts with the addition of ammonia solution (Neon, Suzano, São Paulo, Brazil). Afterwards, the NTs were dispersed in the aforesaid solutions under stirring. Then, the mixture was either stirred or submitted to ultrasonic bath (Prevtech, São Paulo, Brazil) to allow a better dispersion and ions diffusion. Thereafter, the mixture was thoroughly washed with deionized water and a diluted ammonia solution till the pH = 7 to remove the ions physically adsorbed on the NTs surface. Finally, the solids were dried at 80 °C for 2 h. The corresponding metal cations had a metal loading of ca. 1.2 wt%, where CoNT, CrNT, NiNT, AgTNT and AlNT denote the cobalt, chromium, nickel, silver and aluminum incorporated into the NTs, respectively.

The wetness impregnation method was used to incorporate Ag and Pt into the titanate nanotubes by using the corresponding aqueous solution of silver nitrate and hexachloroplatinic acid solutions to produce AgNT and PtNT catalysts, respectively. The theoretical content of the metals was 1.0 wt%. All catalysts were heated under He/oxygen flow, prior to their use.

### 2.3. Characterizations of the Catalysts

X-ray diffraction (XRD) patterns of the solids were recorded on a Shimadzu XRD6000 (Shimadzu, Osaka, Japan) diffractometer, which was equipped with CuKα radiation at 40 kV and 30 mA. The measurements were obtained at wide angles with 2θ values varying from 5 to 70°. The patterns were compared to those of Joint Committee on Powder Diffraction (JCPDS).

The nitrogen adsorption-desorption isotherms were measured using an ASAP 2000 Micromeritics equipment (Micrometrics, Norcross, GA, USA). Prior to the analysis, samples were degassed at 150 °C for 12 h. The specific surface areas were determined by Brunauer–Emmet–Teller (BET) method, and the pore size distributions were calculated by Barret–Joyner–Halenda (BJH) method to the desorption branch of the isotherms.

The morphology of the solids was examined by Scanning electron microscopy measurements in a Quanta-FEG FEI electron microscope equipped with an EDX Link Analytical QX-20,000 system (FEI Quanta, Hillsboro, OR, USA) coupled to the SEM microscope (SEM-EDS). The acceleration voltage used was 2 kV. Previous dispersion of the spent catalysts in an aluminum sample holder and sputtering the catalysts with gold (Sigma-Aldrich, St. Louis, MO, USA) were performed.

Electron paramagnetic resonance (EPR) spectra of the samples were measured by a Bruker spectrometer (Bruker, Rheinstetten, Germany) to detect the local environment of iron atoms and valence states. The EPR measurements were performed at the X-band microwave frequencies close to 9.5 GHz. The apparatus has a double cavity with high frequency modulation of 100 kHz. Before the measurements, the samples were placed in quartz tubes of 4 mm inner diameter at room temperature.

The microstructures of selected titanate nanotubes were observed using transmission electron microscopy (TEM) using a JEOL JEM 2010 microscope (JEOL, Tokyo, Japan) operating at 200 kV. The high-resolution transmission electron microscopy micrographs (HRTEM) of the periodic structures were obtained by the Fourier method. Previously, the samples were prepared by ultrasound dispersing a certain amount of the sample in ethanol (Vetec, São Paulo, Brazil) and then dropping the suspension on a carbon-coated grid. Spent samples were characterized in a Transmission electron microscope from FEI Tecnai 20 G2 (FEI Quanta, Hillsboro, OR, USA) and JEOL JEM-2010 by using the same conditions previously mentioned.

Raman spectra of the solids were recorded on a Bruker Senterra spectrometer equipped with an Olympus BX5 microscope (Bruker, Karlsruhe, Baden-Württemberg, Germany) under ambient conditions. A 532 cm^−1^ laser line was used as the exciting source. The laser beam was focused on the sample surface at an intensity of approximately 10 mW and the focus was 100 times. The measurements were referenced to Si at 521 cm^−1^ with 16 data acquisitions in 100 s. Some measurements were performed on a LabRam Raman spectrometer (HR Horiba Scientific, Tokyo, Japan) using a laser line of 532 nm to obtain the Raman spectra. The excitation source was the 532 nm line with the laser power was set at about 10 mW. The spectral resolution was 4 cm^−1^ using an objective lens of 100 times.

Fourier transform infrared spectroscopy (FTIR) was performed in a Bruker equipment (Bruker, Rheinstetten, Germany) in the range of 4000–400 cm^−1^. Approximately 1 wt% of each sample was dissolved in KBr (Vetec, São Paulo, Brazil), before recording the curves.

The acid sites amounts were determined by temperature programmed of pyridine desorption of pyridine (TPD-Pyridine) followed by thermogravimetry (TG) measurements in a TGA/DSC1 Mettler Toledo equipment (Mettler Toledo, Columbus, OH, USA) coupled to a SDT 2960 from TA Instruments (New Castle, DE, USA). The amounts of the acid sites were calculated according to our previous studies [26].

X-ray photoelectron spectroscopy (XPS) experiments were performed at the PHI VersaProbe II Scanning XPS Microprobe (Minneapolis, MN, USA) in Malaga, Spain. After pre-treatment for surface oxidation, XPS analyses were used for determination the oxidation state of the elements and the detection of surface functional groups of selected spent solids. The incident radiation was monochromatic X-ray Al Kα radiation (200 µm, 52.8 W, 15 kV, 1486.6 eV) with a charge neutralizer. The *C 1s* line of adventitious carbon was used as reference at 284.8 eV. High-resolution spectra were recorded at a given take-off angle of 45° by using a multi-channel hemispherical electron analyzer, which operates in the constant pass energy mode at 29.35 eV. Energy scale was calibrated using Cu *2p_3/2_*, Ag *3d_5/2_*, and Au *4f_7/2_* photoelectron lines at 932.7, 368.2, and 83.95 eV, respectively. The Multipack software version 9.6.0.15 (Physical Eletronics, Chanhassen, MN, USA) was used to analyze in detail the recorded spectra. The obtained spectra were fitted using Gaussian–Lorentzian curves to have more accurate extract binding energies of the different element core levels.

### 2.4. Catalytic Tests in SCR-CO Reaction

The catalytic performances of the samples were examined for the selective catalytic reduction of NO_x_ by CO (CO-SCR). About 150 mg of the catalysts (40−60 mesh) were introduced into the quartz fixed-bed quartz reactor (inner diameter 0.7 cm) at atmospheric pressure. Previously, the catalyst was tightly closed into the reactor and pre-treated by flowing 80 cm^3^·min^−1^ of 10% O_2_/He at 250 °C for 1 h at a rate of 10 °C·min^−1^. Subsequently, the catalysts were purged with helium and finally cooled to 30 °C. Afterwards, the mixture of gases comprising of 500 ppm of NO, 1000 ppm of CO, 10% H_2_O (when used), 50 ppm of SO_2_ (when used) and helium as a balance were introduced into the catalytic bed. The catalytic runs were performed with a GHSV value of 48,600 h^−1^. The conversions of NO_x_ and CO were measured by a NO/NO_2_/NO_x_ electrochemical analyzer from Seitron mold chemistry 400 (Seitron S.p.A, Mussolente, VI, Italy). The experimental test rig was depicted in our previous studies [6,13].

The NO_x_ conversions were calculated according to the equations presented in the references [13], as follows:

(1) $$NO_{x}\;\mathrm{conversion}=\frac{NO_{x\;in}-NO_{x\;\mathrm{out}}}{NO_{x\;\mathrm{in}}}\times 100\%$$

where $\left[NO_{x}\right]=\left[NO_{x}\right]+\left[NO_{2}\right],$ and the subscripts in and out represent the inlet and outlet concentration of NO_x_ at steady state, respectively.

Where NO_in_ refer to the NO_out_ concentrations at the reactor inlet and outlet, respectively.

The turnover frequency (TOF) was determined the number of moles of NO_x_ converted per mole of metal atom per second, according to the previous work [6].

The reaction rate (r) was calculated as follows:

(2) $$r_{NO_{x}}=\frac{{\left[NO_{x}\right]}_{\mathrm{in}}-{\left[NO_{x}\right]}_{\mathrm{out}}}{W_{\mathrm{cat}}\times \mathrm{time}}\;$$

where [NO_x_]_in_ and [NO_x_]_out_ are the concentration of the gas fed in the reactor and concentration of the gas out of the reactor, respectively. w_cat_ represents the catalyst mass per 6 h of reaction time.

## 3. Results and Discussion

### 3.1. Structural Characterizations

**Figure 1:**
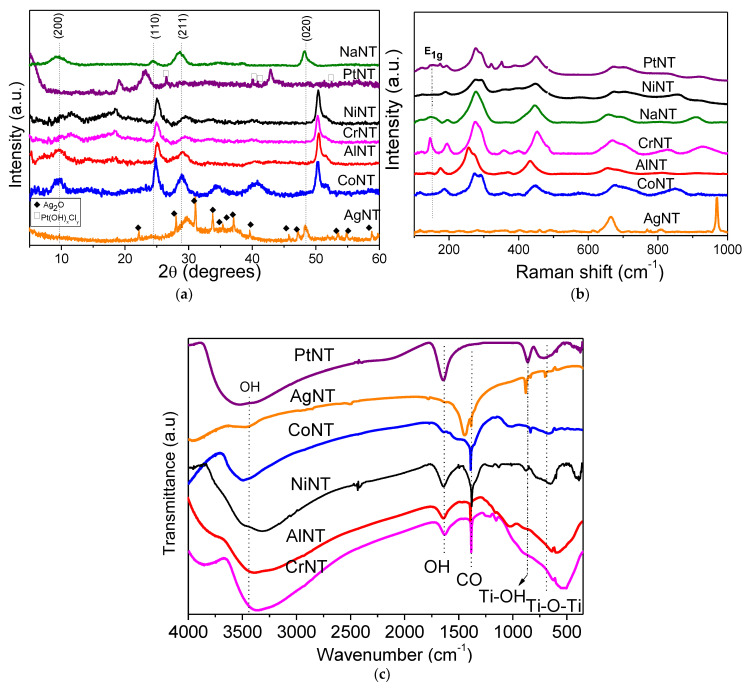
(**a**) XRD diffractograms, (**b**) Raman and (**c**) FTIR spectra of the catalysts studied. The main hkl reflections of Na2Ti3O7 are shown in parenthesis above the diffraction lines.

X-ray diffraction (XRD) patterns of the solids are depicted in Figure 1a. The pristine NaNT shows four diffraction peaks at 2θ equal to 10.1, 24.3, 28.7 and 48.3°, attributed to the 200, 110, 211 and 020 reflection planes, respectively [25,26,27,28,29]. All peaks shown in the NaNT diffractogram can be indexed to the *P21/m* space group from the monoclinic structure of Na_2_Ti_3_O_7_. According to the findings, the Na_2_Ti_3_O_7_ structure has an edge-shared TiO_6_ octahedron in a zigzag-like patterned arrangement, where the Na^+^ ions reside between these layers and occupy two different crystallographic sites bound to the oxygen anions [26,27].

Moreover, the reflection at 2θ = 10.1° (200) is the characteristic peak of the periodic layer structure of the titanate nanotubes possessing an interlayer distance of 0.84 nm (Table 1). Several studies illustrate the layered structure of the titanate and silicates-based materials [4,27]. Compared to NaNT, all solids containing metal impregnated or intercalated into the titanate nanotubes have similar features, being NiNT and AgNT exceptions. Noteworthy, the CoNT experiences a downshift of the diffraction angles of the Na_2_Ti_3_O_7_ phase, which is an indication of the structural disorder of the titanates due to the replacement of the Co metal cations by sodium. Consequently, there is an expansion of the (200) plane favoring the diffusion of the Na+ ions diffusion through the interlayer and facilitates the ion exchange process.

As the layered structure of the titanate nanotubes is composed of the TiO_6_ octahedra, this feature allows Na^+^ cations to diffuse and easily be exchanged by Al^3+^, Cr^3+^ and Ni^2+^ and Co^3+^ ones. Accordingly, the Na/Ti ratios of the MeNT are lower than that of NaNT, which is an indication of the sodium removal of the sodium trititanate structure (Table 1).

Besides, NiNT solid does not show the Na_2_Ti_3_O_7_ tubular structure probably in reason of the fact that part of the Ni^2+^ species might be intercalated into the NTs structure, but most of these species are found in the NiOOH form on the NTs surface. This could be a result of ammonia washing during the synthesis of the solids, in agreement with the findings [29,30]. Moreover, the good dispersion of intercalated Pt nanoparticles (decorated) on the nanotubes shows that the impregnation of Pt on NTs does not significantly affect the titanate structure, implying that the scrolled titanate nanosheets remain. However, the (110) peak in PtNT diffractogram shifts to the left compared with the other catalysts due to the presence of intercalated Pt into the NTs structure. In addition, some characteristic peaks of a minor platinum contribution coming from PtO_x_, PtO_x_Cl_y_ or Pt(OH)_x_Cl_y_ species are observed, as found elsewhere [25,26].

In the case of AgNT, however, the main reflections at 2θ = 32.4 (111), 38.1 (200) and 55.4° (220) correspond to the Ag_2_O cubic structure (space group: *Pn-3m*) [31,32,33]. Besides that, the silver incorporation to the NTs results in a solid with high crystallinity due to the organization of the monoclinic structure of the solid. Moreover, both inner and outer surfaces of NTs have negative charges, which allow the metals to be adsorbed on these surfaces [28,34,35].

Meanwhile, the AlNT catalyst exhibits low intensity peaks at 2θ = 15.4° (020) and 32.1° (120), which could be ascribed to orthorhombic oxy-hydroxide boehmite γ-AlOOH structure (space group *Cmcm*). This result is in agreement with previous findings on boehmite based materials [36]. For CoNT, the peaks at 2θ values of 10.6 (003), 19.6 (006), 35.1 (102) and 39.0° (015) may depict the existence of cobalt oxyhydroxide CoOOH (space group of *R3m*) as a poor crystallized phase [2,37]. Furthermore, all of these extra framework reflections seem to be on the surface of the titanate nanotubes. Therefore, the adsorption of the ions on the outer surfaces is likely whereas that on interlayer is reduced due to the difficulty of the ions to diffuse into the inner surface.

Moreover, CrNT has a very broad peak appearing at 2θ value of 18.4° (003) and another one at 2θ = 12.4° with d = 0.8 nm, which could be indexed to be from the rhombohedral structure of chromium oxyhydroxide α-CrOOH grimaldiite phase (space group: *R-3m*) as found elsewhere [2,38].

Thus, the ion-exchange of Na^+^ (102 pm) ions by a cation possessing lesser ionic radii such as r(Al^3+^) = 53 pm, r(Cr^3+^) = 84 pm or r(Co^3+^) = 61 pm favors the metals incorporation into the NTs structure. This is consistent with the increased interwall distance of the MeNT compared with that of NaNT (Table 1). In contrast, Ag^+^ having a bigger atomic radius of r(Ag^+^) = 115 pm may form an extra framework Ag_2_O oxide. This also suggests that a possible solid-state reaction between Ag_2_O and Na_3_Ti_3_O_7_ may take place.

Raman measurements are performed to investigate the structure of the MeNTs and the electronic interaction between Ti and metals. The region of the Raman spectrum that corresponds to the lattice vibrations is shown in Figure 1b. Accordingly, NaNT has vibrational modes at around 158 (E1g), 195 (Eg), 276 (Ag), 445 (B1g), 656 (A1g), 696 (Ag) and 908 (Bg) cm^−1^ [25]. The weak bands at 156 and 192 cm^−1^ are attributed to the anatase-type structure of TiO_2_, whereas the modes at 276 and 656 cm^−1^ are due to the Na cation coordinated to the oxygen atom of the framework Ti–O vibration [25,35]. At 445 cm^−1^, the Ti–O–Ti framework vibration is clearly visible [35]. Importantly, the band at 908 cm^−1^ has previously been correlated with the symmetric stretching vibration mode of a short Ti–O bond of sodium titanate in layer structure and the Ti–O–Na stretching vibration in the interlayer regions of the nanotube walls [25,34].

The spectrum of AlNT is very similar to that of NaNT, but a high downshift of the main titanate nanotubes vibrational modes confirm the structural disorder of the solid due to the Al insertion in the NTs structure. Moreover, an absorption band at around 360 cm^−1^ evidences the presence of the α-AlOOH particles [36]. Important is to mention that the band associated with the Ag mode for AlNT splits into two shoulders at 256 and 275 cm^−1^, having these modes much lower intensities than those of the original Ag ones. This indicates that the replacement of the Na cation coordinated to the oxygen atom of the framework Ti–O vibration by Al one provokes a hardening of the vibrational modes. These observations are consistent with the XRD results. The modes of CoNT spectrum upshifts comparing with that of NaNT, which indicates the softening of the modes, as Co is incorporated to the NTs structure. Similar to AlNT, the Ag modes split into two new ones illustrating the perturbation of the Ti–O bond by cobalt incorporation. Besides, the appearance of the bands with maxima at 357, 406 and 582 cm^−1^ is assigned to the presence of α-CoOOH, as found elsewhere [37].

Interestingly, the modes of the titanates nanotubes are not affected by the presence of Cr in CrNT compared to NaNT, confirming that the Cr^3+^ ion exchange by Na^+^ ions, in line with the XRD results. In other words, the titanate nanotubes bands remain virtually unperturbed upon Cr insertion. Moreover, the Raman bands of CrNT become more intense and additional modes at 361, 397 and 827 cm^−1^ are due to the ν_1_ Cr–O symmetric stretching mode in the α-CrO(OH) structure [38]. Upon the Ag addition to the NTs, the vibrational properties of the AgNT sample change substantially, as shown in Figure 1b. The titanate nanotubes bands decreased in intensity and some of them vanished from the spectrum, implying that the phase transformation of the titanate nanotube structure occurs after the silver addition. This feature indicates that silver does not participate in the bonds included in the vibrations associated with titanate nanotubes bands, most probably in the formation of the Ag_2_O phase. Based on XRD results, the presence of Ag_2_O confirms the crystallization of the AgNT catalyst. Indeed, the modes located at about 561, 700 and 1010 cm^−1^ are associated to the Ag–O mode vibration, as reported in the literature [33].

The Raman spectrum of PtNT exhibits broad bands characteristic of the tubular nanotube with the layered structure of the NTs. Some of these bands depict a blueshift of the modes near 630 cm^−1^ due to the Me–O bonds, as a consequence of the intercalation of the ions in the NTs structure. The findings also state that the observed blueshift can be associated with the creation of oxygen vacancies in the NTs structure [8]. Moreover, two narrow bands appear at around 350–400 cm^−1^, which can be ascribed to the formation of the PtO_x_, PtO_x_Cl_y_ and Pt(OH)_x_Cl_y_ phases [26].

FTIR spectroscopic investigation of the as-synthesized catalysts (Figure 1c) show the strong absorption band at 3600 cm^−1^ corresponding to the stretching vibrations of the OH groups from the Ti–OH bonds of the trititanate structure [39,40]. Moreover, these OH groups can be attributed to either physically adsorbed water or the extra framework hydroxides such as α-CrOOH, α-CoOOH, NiOOH, α-AlOOH and Pt species, in agreement with XRD and Raman results. Most likely, the low concentration of OH groups of AgNT is illustrated by the small size of the aforesaid absorption band, which is evident from the XRD measurements. At 1620 cm^−1^, the weak infrared bands with bending vibrations are assigned to the OH groups in all solids [39]. It should be pointed out that the band at 1360–1400 cm^−1^ arises from C–O bonds of surface carbonate species during the preparation of the solids. The presence of these surface carbonates will be confirmed later by XPS. Meanwhile, the FTIR spectra depict bands at around 707, 930 and 990 cm^−1^, being characteristic of the Ti–OH and Ti–O–Ti bonds from the NTs lattice vibrations [18,39]. Moreover, these bands below 500 cm^−1^ can be attributed the Me–O vibrations.

### 3.2. Morphology and Textural Properties of the Catalysts

**Figure 2:**
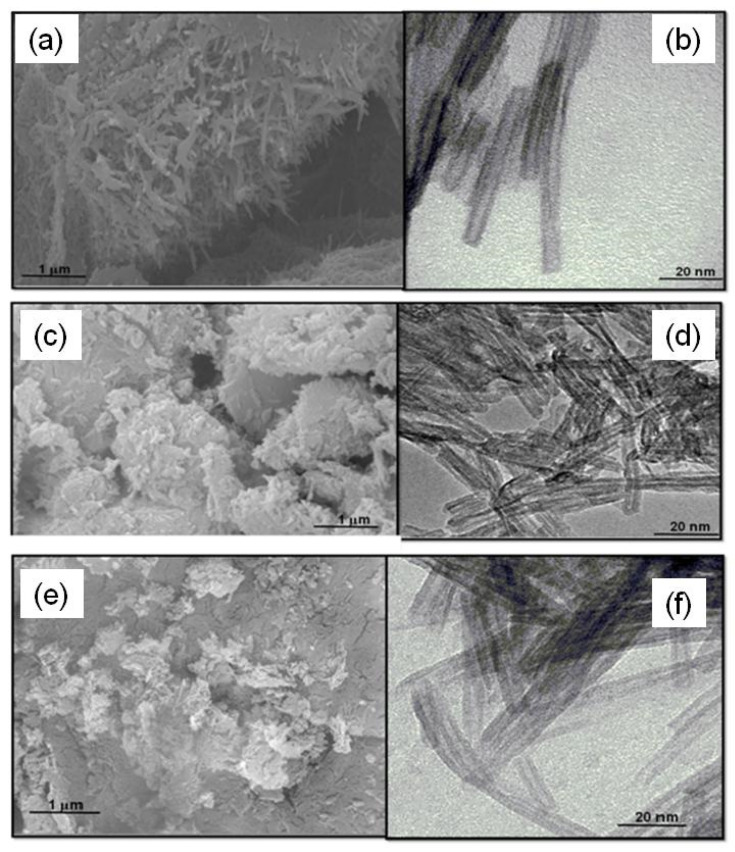
SEM-EDS and TEM micrographs of the catalysts studied: (**a**,**b**) NaNT, (**c**,**d**) CoNT, (**e**,**f**) CrNT.

The surface morphology of the catalysts is examined by SEM micrographs. Appearance of abundant nanorods mostly agglomerated is illustrated in the SEM micrograph of NaNT (Figure 2a). The EDS spectrum illustrates that the sodium, oxygen and titanium contents on the surface are, respectively, 0.45, 46.7 and 28.7%. This indicates that the hydrothermal preparation method allowed the formation of the Na_2_Ti_3_O_7_ phase, taking into account the XRD and Raman results. Besides, the HRTEM micrograph (Figure 2b) confirms the existence of the randomly tangled nanotubes with inner empty structure and diameters varying of 3–10 nm and several hundred nanometers of length. These morphological aspects of the NTs are predictable by literature reports using similar hydrothermal synthesis methods [23,35].

The SEM micrograph of CoNT consists of disordered agglomerates of particles in the form of platelets (Figure 2c), although some nanotubes are visible in the edge of the plates. Taking into account the expectations that the ion-exchange of Na by Co results in the substitution of the sodium ions in the NTs structure, the EDS spectrum illustrates that a very low amount of Co ca. 0.7% is on solid surface. Moreover, Co is uniformly distributed throughout the solid surface. This is consistent with the Raman spectrum of the solids because it also indicates that the displacement of the bands caused by Co insertion in the NTs structure. Additionally, the CoNT morphology exhibits entangled nanotubular shaped particles with some black dots representing the Co nanoparticles decorating the nanotubes, as observed by HRTEM micrograph (Figure 2d). The measured interwall distance is 0.91 nm corresponding to the NTs, demonstrating the successful incorporation of Co into the NTs structure.

The ion-exchange process of replacing of Na^+^ ions by Cr^3+^ gives rise to agglomerated particles of irregular morphology, but some nanorods remain visible (Figure 2e). Moreover, the EDS spectrum suggests that the Cr content is 1.4% whereas the Na one is lesser than the one in NaNT, in agreement with the low Na/Ti ratio observed in Table 1. Most likely, the ion-exchange process does not affect the morphology of the solids with CrNT displaying entangled titanate nanotubes formation; however, the length of CrNT becomes short (Figure 2f).

**Figure 3:**
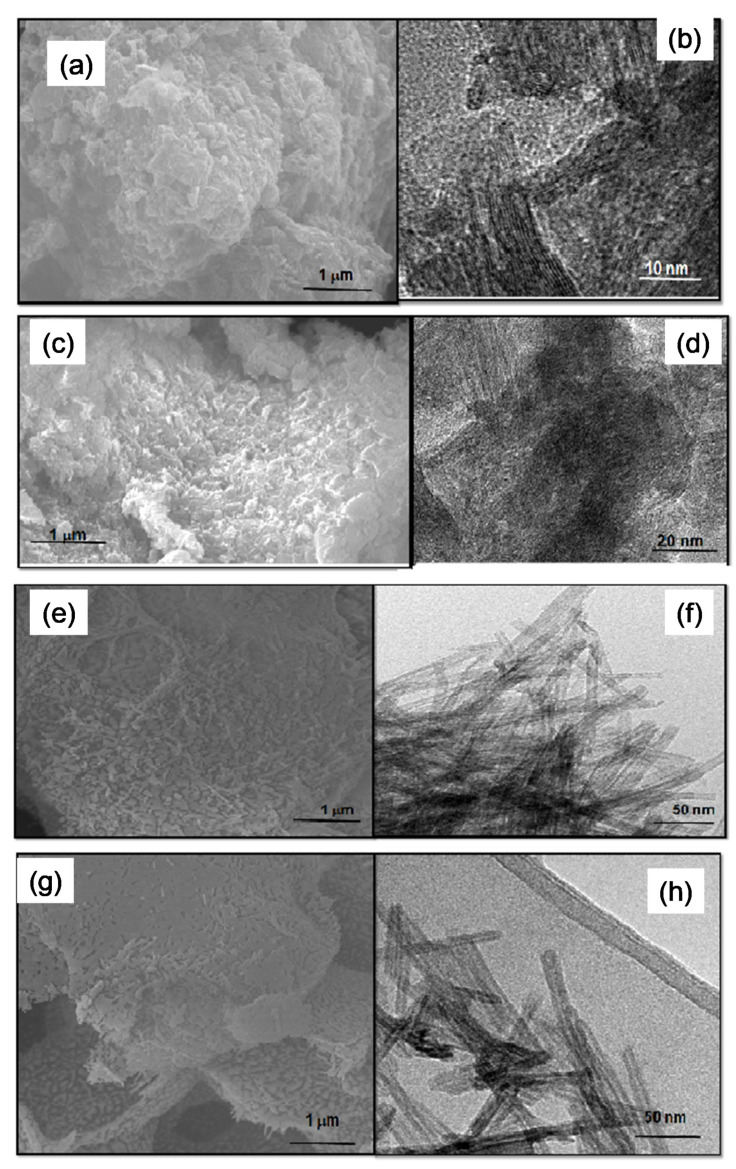
SEM-EDS and TEM micrographs of the catalysts studied: (**a**,**b**) AlNT, (**c**,**d**) AgNT, (**e**,**f**) NiNT and (**g**,**h**) PtNT. The included Figure in TEM image of PtNT is related to the Pt nanoparticles.

AlNT shows particles much less morphologically similar to those observed in CrNT, with irregular shapes and the ensemble of nanotubes forming voids between the particles (Figure 3a). This could be an indication of the formation of large pores surrounded the particles, which is later confirmed by textural properties analyses. Although a certain amount of Na^+^ ions is substituted by Al^3+^ ones in the NTs structure with the consequent drop of the sodium content, the EDS spectrum reveals that most of the Al content of ca. 1.8% is concentrated on the solid surface. Accordingly, Raman measurements suggest that the structural disorder of the AlNT due to the Al insertion in the NT structure and the presence of the α-AlOOH phase. In particular, HRTEM micrograph of AlNT displays the interlayer distance of the ca. 0.95 nm pointing to the NTs structure along with the nanoparticles (black dots in Figure 3b) attributed to being boehmite.

Conversely, the SEM micrograph of the AgNT particles significantly differs from those of the other MeNTs. The formation of aggregated particles with a rough surface (Figure 3c) indicates that these particles are visually bigger than the metal-containing NTs counterparts. Moreover, small voids are observed among the particles representing the shrinkage of the NTs pores, as further seen by textural properties analyses. Moreover, the EDS spectrum confirms the presence of 0.57% of titanium along with 12.0% of Na, 54.6% of Ag and 24.0% of O atoms, respectively. This indicates that silver species are entirely deposited on the nanotubes surfaces and some nanosheets can be formed, as previously indicated by XRD and Raman results. Importantly, the residual sodium content is not negligible as the Ag ions deposited on solid surface. HRTEM micrograph of AGNT is seen in Figure 3d. The image clearly depicts big particles with a lattice spacing of 0.23 nm corresponding to the (200) plane of Ag_2_O, as observed by XRD. These big Ag_2_O particles are deposited on the solid surface, but the lattice fringes of the (001) and (020) plane highlight that the monoclinic titanate nanotubes structure remains in the solid, after the ion-exchange process. The lattice fringe of 0.24 nm corresponding to the (111) plane of Ag_2_O is clearly visible.

The SEM images of NiNT are illustrated in Figure 3e,f whereas PtNT micrographs are seen in Figure 3g,h. As it can be seen, the morphology of the PtNT and NiNT surfaces is very similar to that of NaNT (Figure 2a). Nevertheless, the densification of the nanotubes can be clearly seen forming plates, which demonstrates that the ionic exchange of Na by Ni and Pt promotes the aggregation of the tubular morphology in a clumped manner. Notably, the Ni/Ti and Pt/Ti ratios measured by EDS are 0.17 and 0.18, respectively. This implies that most of the Na ions (Na/Ti ratio of NaNT is 0.22) are removed during the ionic exchange and impregnation processes, and some Pt and Ni ions are substituted by sodium ions in the layered structure of the PtNT and NiNT.

Furthermore, TEM image of NiNT clearly illustrates that even after the ionic exchange, the tubular morphology is preserved (Figure 3f), being the bundles of nanotubes entangled with Ni addition. Moreover, the morphology of the PtNT indicates that the tubular multiple-walled like morphology is still retained, but with their sizes are reduced upon Pt incorporation (Figure 3h).

Moreover, the Ni and Pt nanoparticles are not observable in the framework of the tubular structure indicating that they are well dispersed on both inner and outer surfaces of the nanotubes due to the electrostatic interaction between the negative charges of the NTs sheets and positively charged cations [29]. Important is to say that the nanoparticles cannot be seen due to the low resolution of the images.

These results are consistent with the XRD, Raman and FTIR measurements that suggest the maintenance of titanate nanotubes layered structures after ion exchange. On the contrary, the NiNT, PtNT and AgNT are exceptions, since their morphology is not similar to that of NaNT.

**Figure 4:**
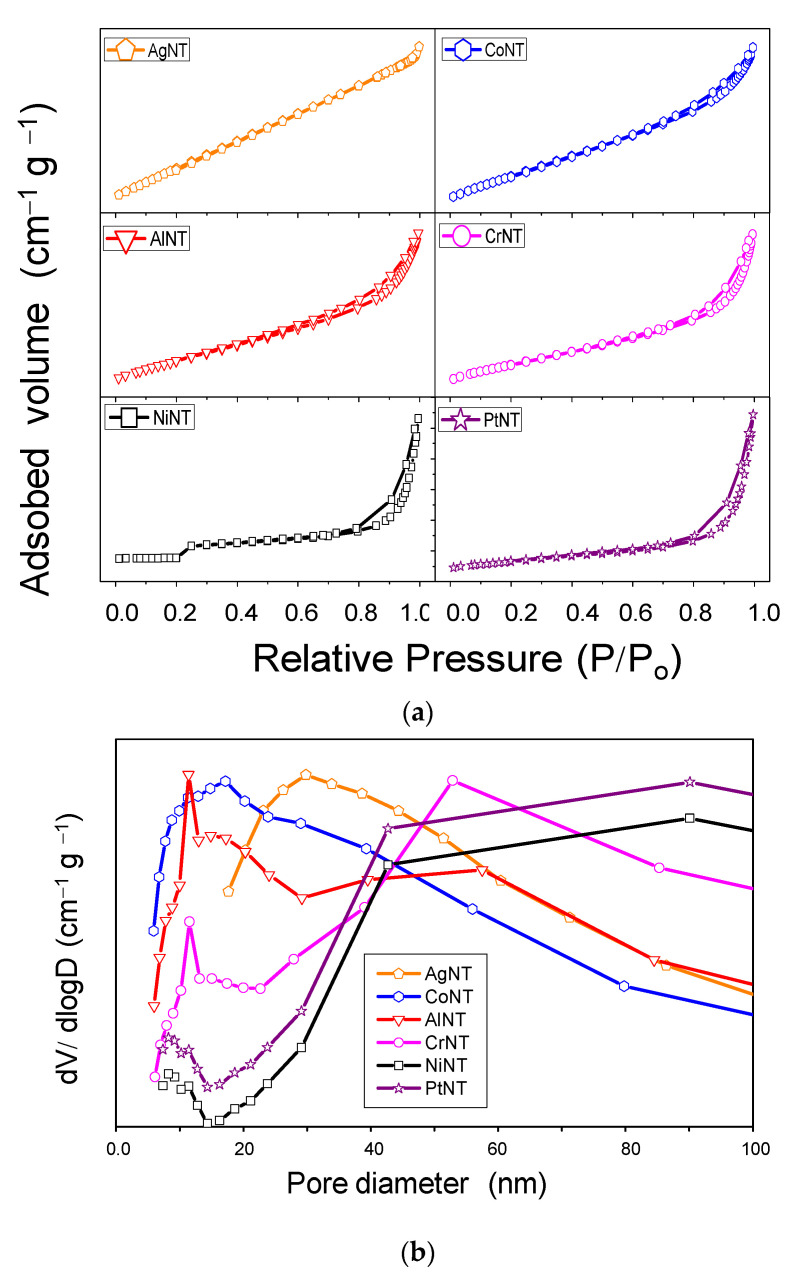
(**a**) N_2_ adsorption-desorption isotherms and (**b**) BJH pore size distributions of the catalysts.

Nitrogen physisorption curves are assigned to be from type IV isotherms, which is characteristic of mesoporous materials possessing slit-shaped mesopores (Figure 4a). The H3 hysteresis loops of CrNT, AlNT, NiNT, PtNT and CoNT start at high relative pressure. It can indicate a remarkable porosity of the solids revealing large pore sizes and their pore connectivity, as well. This is reasonable to expect since the findings accord that the isotherms of sodic titanates nanotubes are similar to those of the metal-containing titanate nanotubes [2,19].

On the contrary, the appearance of an H4 hysteresis loop for AgNT suggests that the solid has large particles compared to the other MeNT, which is consistent with previous results of SEM-EDS, XRD, TEM and Raman spectroscopy. The BET surface area of NaNT is 189 m^2^·g^−1^ and the pore volume is of 0.62 cm^3^·g^−1^, as shown in Table 2. The textural properties of the MeNT depict significant quantitative differences in the parameters, when the Me are incorporated into NTs structures. Especially for CoNT, NiNT and AlNT, the surface area and pore volume become much higher than that of NaNT, as a result of the increased interwall distance (Table 1) experienced by the introduction of Al, Ni and Co into the interlayer region.

The results in Table 2 also illustrate a trend of declining of the textural properties values for PtNT, CrNT and AgNT compared to the Ni, Al and Co substituted titanate nanotubes. This result can be correlated to the formation of nanoparticles on the solid surfaces of PtNT and CrNT.

For AgNT, the deposition of the Ag_2_O on the NTs surface for the solid might be caused by the strong electronic interactions of the bigger Ag particles and the nanotubes surface rather than the Ag ions bound in the NTs lattices.

Furthermore, the CoNT, AlNT and CrNT have considerably higher pore volume values than that of the NaNT indicating the expansion of the pores due to the metal incorporation into the inner and outer surfaces of the NTs structures. Moreover, all the pore size distribution curves exhibit broad features that can be reasoned as the presence of large mesopores (Figure 4b). Among the investigated solids, the deposition of the silver oxide particles mainly affects the pores with their consequent blocking and formation of micropores, in line with SEM-EDS and TEM results.

### 3.3. Electronic States, Reduction Behavior and Acidity of the Catalysts

**Figure 5:**
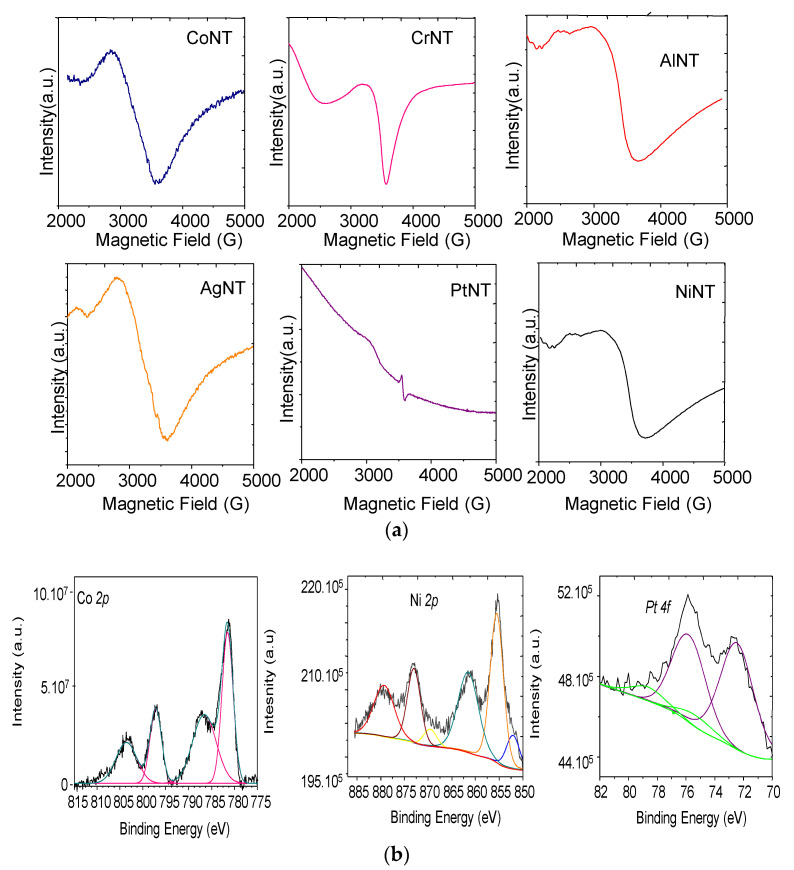
(**a**) EPR spectra of the studied of catalysts. (**b**) XPS spectra of the Co 2*p*, Ni 2*p* and Pt 4*f* core level, respectively, for CoNT, NiNT and PtNT.

Electron Paramagnetic Resonance (EPR) spectroscopy is a standard and non-destructive tool to determine the valence state of some elements of the studied catalysts. The EPR spectra of the samples exhibit two asymmetric signals centered at 2000 and 3600 G, which is typical for metal oxides paramagnetic species. Despite the fact that the EPR spectra of titanate nanotubes do not differ in shape, there are considerable differences among them. For instance, EPR line of CoNT is much broader than that of CrNT (Figure 5a) probably due to the local atomic and electronic structure of Cr in the NTs and interaction mechanisms between spins, as well. Indeed, the broad line shape of CrNT depicts the well asymmetrical defined doublet with *g* value of 5.04. It is found that the paramagnetic lines of the EPR spectrum can be undoubtedly assigned to Cr^3+^ species (r = 0.61 Å) partially occupying the positions of Ti^4+^ in the NTs lattice.

For the sake of comparison, trivalent chromium species in a structurally related Cr-doped TiO_2_ solid shows high spin configuration where a substitutional Cr^3+^ species occupies the vacant tetragonal Ti^4+^ sites environment, which are coordinated by a slightly distorted octahedron of oxygen [41]. This is corroborated by Raman results, demonstrating the existence of Cr in the NTs framework and CrOOH species.

The Ti^3+^ species from NTs are observable through the resonance signal with g values close to 2.0, as found elsewhere [42,43]. Moreover, the paramagnetic defects of the foreign species are embedded in the titanate nanotubes structure possessing a lower symmetry [44]. For, AgNT (Figure 5a), two isotropic bands doublet signals at around 2500 and 3200 G are ascribable to the isolated Ag^+^ ions and Ag coordinated to oxygen on the NTs surface [45].

For CoNT, the Co^3+^ ions are included into the monoclinic layered trititanate structure as a result of the Na^+^ substitution in the interlayer region. The *g* value of 4.9–5.4 indicates that the solid contains Co^2+^ and Co^3+^ species. On the bases of the findings reported so far, Co^2+^ can be incorporated in the framework of trititanate nanotubes occupying octahedral sites and substituting partially the Ti ^4+^ions, as well [46,47]. Although the differences in the atomic radius of Co^2+^, e.g., r = 0.745 Å and Co^3+^, e.g., r = 0.61 Å when replacing the Ti^4+^, e.g., r = 0.605 Å into the TiO_6_ octahedra exists, it is believed that the trivalent one may be visibly incorporated in the NTs framework due to its size being close to that of titanium. In agreement, EDS indicates the surface of the solid has Ti and O in trivial amount besides the Co incorporated in the NTs structure.

The EPR results of AlNT and NiNT depict similar features to those of CoNT with asymmetric signals associated with isolated Me ions. Especially for PtNT, the EPR spectrum has narrow anisotropic EPR signals, which is attributed to the Pt species in accordance with the Pt/TiO_2_ samples [48]. The reports also show that the oxygen vacancies of TiO_2_ surfaces are identified with *g* values close to 2.0 which is in agreement with our XRD results.

**Figure A2:**
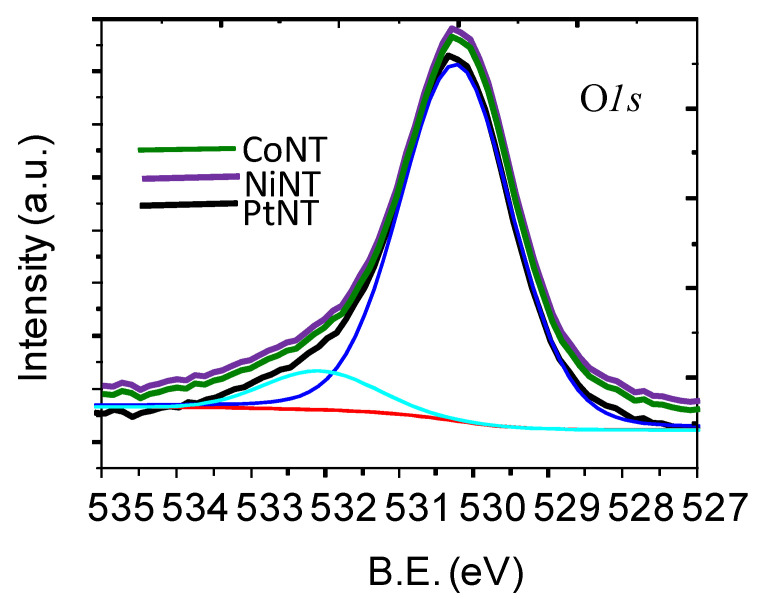
O 1*s* core level XPS spectra for fresh titanate nanotubes.

Table 3 summarizes the results of the XPS, data of selected samples. On the basis of the XPS results, all the studied titanate nanotubes possess Ti^4+^ on solid surface. The Ti 2*p* core level spectra display a doublet Ti 2*p*_3/2_ and Ti 2*p*_1/2_ at two main peaks at 458.7 and 465.1 eV, respectively, typical of Ti^4+^ in TiO_2_. According to previous literature on titanate nanotubes, these values were also observed for the walls of Ti_3_O_7_^2−^ trititanate structure with cations intercalated [4,26]. All of the solids have almost identical binding energies for Ti *2p* core level. Moreover, the remaining sodium content still visible on the surface of the NTs with Binding Energy (BE) for Na *1s* being 1071.4–1071.6 eV form the remaining Na^+^ bonded to titanate structure (Table 3), in agreement with EDS analyses. Notably, the O 1*s* signals have 91% of the contribution (Figure A2, Appendix A) appearing at 530.3 eV. This signal is assigned to lattice oxygen in the NTs studied, which is indicative of either lattice oxygen of TiO_2_ or sodium trititanate structure, in line with the literature reports [25,26]. Moreover, the minor contribution arises at 532.2 eV, which suggests the presence of the oxygen from OH groups of the NTs and the extra framework NiOOH, CoOOH, Pt(OH)Cl_x_ species as shown in NiNT, CoNT and PtNT, respectively. This agrees with the FTIR spectra that showed the presence of OH groups.

For CoNT samples, the Co 2*p* core level has two broad peaks (Figure 5b) illustrating the typical Co 2*p*_3/2_–Cr 2*p*_1/2_ doublet. The binding energy values for the Co 2*p*_3/2_ and 2*p*_1/2_ core levels are at around 781.0 and 796.0 eV assigning the presence of Co^2+^ in tetrahedral coordination state and shake-up satellites, respectively. However, the presence of Co^3+^ species cannot be ruled out [13,25]. The satellite indeed indicates the Co^2+^ greatly interacting with OH groups from CoOOH phase. This is in accordance with XRD and Raman EPR results of the solids.

The Ni 2*p* core level spectrum for NiNT presents the main Ni 2*p*_3/2_ peak at 855.9 eV corresponding to the Ni^2+^ state. This indicates that the structure of the trititanate nanotubes is preserved with Ni intercalated into the titanate interlayer. The Ni 2*p*_3/2_ shake up satellite peak 861.2 eV suggests the presence of Ni^2+^ in NiOOH, as observed by XRD and EPR results.

The Pt 4*f* core level spectrum for the PtNT sample exhibits an asymmetric Pt 4f_7/2_ peak that can be decomposed into two contributions at 72.5 eV (88%) and 75.3 eV (12%), as shown in Figure 5b. The components at 72.5 and 75.3 eV arise from Pt^2+^ and Pt^4+^ species, respectively, in either Pt oxides or Pt(OH)Cl_x_ form [13,25,49]. More importantly, the Cl *2p* core level spectrum for PtNT shows the doublet Cl 2*p*_3/2_ and Cl 2*p*_1/2_ at 199.0 and 200.6 eV, respectively; this is typical of Pt(OH)Cl_x_ species [13,49].

The presence of Ni^2+^ ions as intercalated nickel species is confirmed in NiNT spectrum whereas PtNT shows Pt species in two valence state such as Pt^2+^ and Pt^4+^ ions from PtO and Pt(OH)Cl_x_, respectively. In the case of CoNT, the Co^2+^ species are found by XPS. This is expected taking into consideration the ion-exchange and impregnation processes used to obtain the titanate nanotubes.

The TPR measurements of fresh NiNT, PtNT and CoNT were previously published [4,25]. As a summary in Table 4, the TPR pattern of NiNT depicts a broad signal in the 400–600 °C range, which is associated with NiO strongly interacting with the NTs support during the reduction of the solid, as shown previously [4]. Contrary, the CoNT exhibits a less reducible species at temperatures as low as 200 °C. This indicates that some Co^2+^ species are not incorporated into the NTs and low interacting with the NTs support whereas the peak at high temperatures suggests the reduction of the Co nanoparticles highly dispersed on solid surface forming the surface like Co_3_O_4_ phase.

In the case of TPR curve of PtNT, two reduction stages are observed at 200 °C and up to 700 °C, most probably due to the reduction of the oxychlorinated platinum species. Presumably, the second stage can be attributed to the consecutive reduction of the [PtO_x_Cl_y_] or PtCl_6_^−^ to PtO_x_ and the further reduction of these species, as found elsewhere [25,26].

The acidity measurements by TPD-pyridine reveals that the amount of the acid sites in NaNT is about 7 μmol Py gcat^−1^ mostly having weak acidity. Contrary, PtNT has the acid sites of medium to strong strength possessing the amount of ca. 261 μmol Py gcat^−1^ (Table 4). This is attributed to the surface Pt species such as oxidized PtO_x_ (PtO or PtO_2_) and chlorine (Pt(OH)_x_Cl_y_ and PtO_x_Cl_y_) species [4,25]. When comparing PtNT and NiNT catalysts, the amount of acid sites decreases with NiNT having the minor amount of acid sites due to the weak acidity of the Ni^2+^ species. Thus, acidity measurements by pyridine-TPD follows the order: CoNT < NiNT < PtNT.

The basicity of the fresh titanates based-solids is investigated by the CO_2_-TPD. For CoNT, the first band is located at 100–300 °C (Table 4) being related to the decomposition of the as-synthesized titanate nanotubes into TiO_2_ species, and possibly the adsorption of CO_2_ on the weak acid sites of the samples. The second peak spans from 300 to 500 °C being related to the CO_2_ adsorption on the medium-strength basic sites, arising from NaO_x_ species TiO_2_ or CoTiO_3_ phases [25]. The NiNT catalyst has low intensity desorption peak at temperatures as low as 300 °C due to the decomposition of the titanate and the formation of weak basic sites, as well. At temperatures superior to 300 °C, the medium strength basic sites are observed accounting for the low interaction of Ti with NiO. In the case of PtNT, a peak in the 100–250 °C range is due to NaO_x_ presence and the nanotubes decomposition [4]. The second peak arises in the 300–500 °C range assigning to the CO_2_ adsorption on weak Pt or Ti sites.

All these facts are evidenced by the EPR, XRD and Raman measurements of the solids shown in the previous section.

### 3.4. Catalytic Results

**Figure 6:**
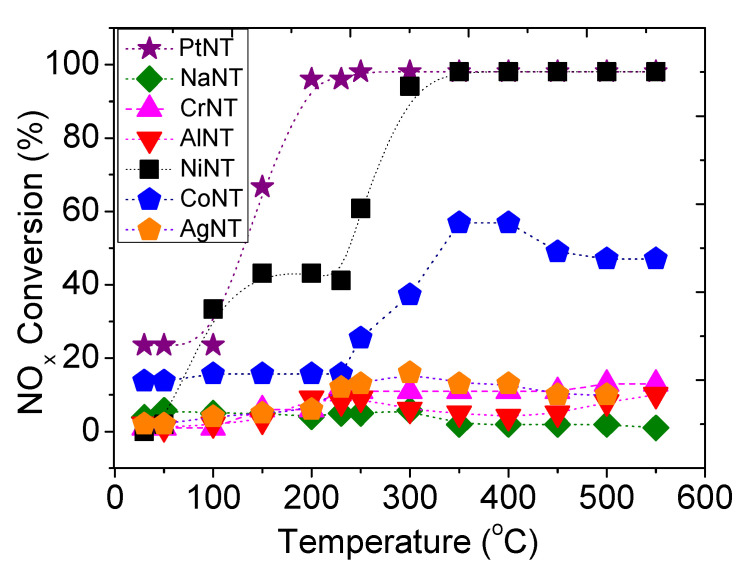
NO_x_ conversions of the titanate nanotubes as a function of the temperature during CO-SCR reaction. Reaction conditions: amount of the catalyst, 0.15 g; reaction mixture, 500 ppm of NO, 1000 ppm of CO and balance in He; flow rate, 80 mL·min^−1^; space velocity, 48,000 h^−1^.

The catalytic evaluation of the solids in the selective reduction of NO_x_ by CO (CO-SCR) reaction is carried out in the temperature range of 50−550 °C. At temperatures lower than 100 °C, NO_x_ conversions inferior to 37% are seen for all titanate nanotubes catalysts (Figure 6). Importantly, preliminary investigations without using catalysts in blank runs afforded less than 5% of conversion at 100 °C. As NO_x_ can be physically adsorbed on the surface of the solids at low temperatures [6,48], the subsequent increase of the activity is observed at higher temperatures.

The NaNT sample possessing Ti^4+^ centers from Na_2_Ti_3_O_7_ phase is inactive in the reaction, independently of the temperature evaluated. As the solids have residual alkali sodium contents, it can be seen that the presence of Na does not affect the catalytic performance during the CO-SCR reaction. In contrast to NaNT, the metal-containing titanate nanotubes are actives in the reaction, suggesting that the metals incorporated in the NTs work as active sites for the acid-base or redox reaction. It is noteworthy that AgNT, AlNT and CrNT exhibit similar behavior with conversions below 15% in all temperature range. Moreover, literature reports show that the catalyst having sites for NO_x_ adsorption on the alkali elements such as Na, protonic nanotubes and alkaline earth are active in the reaction [50,51,52,53]_,_ but the presence of a metal is needed.

Further reaction temperature increase from 100–250 °C gives distinct behavior for MeNTs solids studied. The literature reports show that the CO-SCR reaction initially proceeds by the simultaneous oxidation of NO to surface nitrates as strong oxidants and oxidation of the CO to surface oxygenates [14,15]. This next step is the reaction between these surface intermediates that leads to the formation of NCO and CN species, and finally, the latter species are converted into N_2_; the CO is oxidized to CO_2_ at relatively mild temperatures. Interestingly, Figure 6 shows that NO_x_ decomposition of all the abovementioned MeNTs is very low at temperatures as low as 200 °C, with CoNT, PtNT and NiNT being exceptions. As the reaction proceeds, the catalytic activities suddenly depict a huge upward trend for CoNT, PtNT and NiNT, when the reaction temperature rises above 250 °C. For instance, the NO_x_ conversion on PtNT is 25% at 100 °C and reaches 92% at 200 °C indicating the moderate temperatures causes an improvement of the catalytic properties. Contrary, the activities of AgNT, AlNT and CrNT achieve a plateau with low conversions below 10% in all temperature ranges. 

Importantly, the methods of salt addition to nanotubes, i.e., ion exchange and wet impregnation affect the catalytic performance of the solids. Even though PtNT is prepared via wet impregnation method, the surface acidities of both PtO_x_ and chlorined Pt entities on the tubular NTs play an important role on the catalytic activity, especially the strong adsorption capability of Pt sites toward CO and NO_x_ reactants at low temperatures.

Subsequent temperature increase from 250 to 550 °C results in CoNT, PtNT and NiNT conversions close to 55% at 550 °C, in opposite to NaNT, AlNT, AgNT and CrNT that experience poor performance. Taking into account the presence of the nanotube structures possessing the Na_2_Ti_3_O_7_ phase in these solids (identified later by XRD and Raman of the spent samples), it can be inferred that the sodic phase is thermally transformed to transition states among the trititanate, anatase TiO_2_ and traces of rutile TiO_2_ phases at about 400 °C [25].

Besides, the catalytic behavior of AlNT is apparently different from that of NaNT with NO_x_ conversion of 6% at 550 °C. Such a behavior is considered to be caused by the Al centers incorporated to the titanate nanotubes that promote the CO-SCR reaction. However, a large amount of Na ions on solid surface (Table 1) leads to lower accessibility of the Al species, thus resulting in a very low performance of the AlNT. Notably, the AlOOH phase onto the NTs surface does not improve the catalytic performance of the solid. To verify this assumption, XRD, FTIR and Raman of the spent solids demonstrate that most of the Na ions remain on AlNT surface, while boehmite is deposited on the solid surface.

CrNT has a NO_x_ conversion of ca. 15%, which is considered to be close to that of AlNT and AgNT. Despite the fact that both AlNT and CrNT display almost identical interplanar distance, most of the Cr^3+^ ions are out of NTs lattice forming the well disperse CrOOH oxide. Thus, more Cr^3+^ appears somewhat exposed on surface, as observed from the results of XRD, SEM-EDS, EPR and Raman spectroscopy. It is interesting to note that these trivalent chromium species in extra lattices are assumed to be responsible for the modest catalytic performance of the solids, in comparison with the AgNT, and AlNTs analogues.

It is worthy to mention that a NO_x_ conversion of 7% is accomplished by AgNT, which is indeed two times lesser than that observed in the case of CoNT. The exposition of the Ag_2_O nanoparticles low interacting with the NTs surface (SEM, HRTEM and XRD) may result in a boost of the catalytic performance of the solid. These data nicely demonstrate that the Ag^+^ species located on solid surface assists interacting with the CO and NO_x_ molecules; thus, Ag species are not able to maintain the catalytic performance.

The presence of Co incorporated into the NTs structure depicts a significant influence on the activity of the CoNT catalyst. Even with a small amount of cobalt, e.g., 1.0 wt%, the NO_x_ conversion is noticeably showing a clear increase of two times more than that of AlNT and AgNT. The entire inclusion of Co into the NT structure produces Co^3+^ sites accessible to the reactants and plays an important role in improving the NO_x_ conversion. Conversely, the interaction of Co active sites is inefficient to retain the conversion level. Thereby, the modest performance of CoNT toward NO_x_ and CO is found. In reference to the literature for other Co-based catalysts for SCR reactions [54], the CH_4_-SCR studies on Co-based zeolites demonstrate the thermodynamic equilibrium allows high NO conversions to NO_2_ by O_2_ below 400 °C, although the catalyst is needed to the reaction occurrence.

Furthermore, the catalytic performance of the NTs is greatly improved when nickel is incorporated to the NTs. In line with expectations, note that the beneficial effects of the Ni phase on NTs at around 300 °C are those of increasing the dispersion of the titanate nanotube (further shown by TEM and FTIR measurements of the spent solids) and promoting the reduction of Ni^2+^ sites during the range of temperature studied (Table 4, TPR results). Most probably, the reaction environment provides the reduction of Ni species on the defect sites of the NTs, facilitating the coordination of NO_x_ or CO molecule to a Ni Lewis acid sites and thus improving the catalytic performance. In contrast, isolated NiOOH alone and those incorporated to the NTs sites in NiNT have low acidity (Table 4, acidity measurements) implying in the low efficiency of these Ni sites to convert NO_x_ at temperatures lower than 200 °C (Figure 6). In line with these results, the Ni^2+^ is reduced above 550 °C, facilitating the transformation of the Na_2_Ti_3_O_7_ and NiOOH phases to anatase and their corresponding NiO sites to increase the catalytic performance.

Notably, PtNT reaches the maximum values of NO_x_ conversion at temperatures superior to 200 °C followed by NiNT. The surface acidities of the well dispersed PtO_x_ and chlorinated Pt entities on the tubular NTs (latter seen by TEM) give the highest acidity for the former, as shown in Table 3. Thereby, this results in high NO_x_ conversion within lower temperature region. On the basis CO-TPSR lean trap NO_x_ measurements for Pt-based samples, the CO reacts with NO_x_ forming CO_2_ and NO, along with trace amounts of N_2_ above 330 °C simultaneously [3]. Moreover, our previous studies on optimizing the selective catalytic reduction of NO by CO reaction in the presence of distinct oxygen concentrations have demonstrated that the catalysts are more tolerant to the presence of oxygen in concentrations as low as 1000 ppm, which inhibit the oxidation of the CO by oxygen [55]. In agreement, the CO_2_-TPD profile indicates that the PtNT catalyst could avoid the oxidation of CO, since the Pt sites have a low affinity for this molecule maintaining the CO as a reducing agent to react with NO_x_ during the CO-SCR reaction.

#### Effect of the SO_2_ and Water Vapor Poisons on the Catalytic Performance

**Figure 7:**
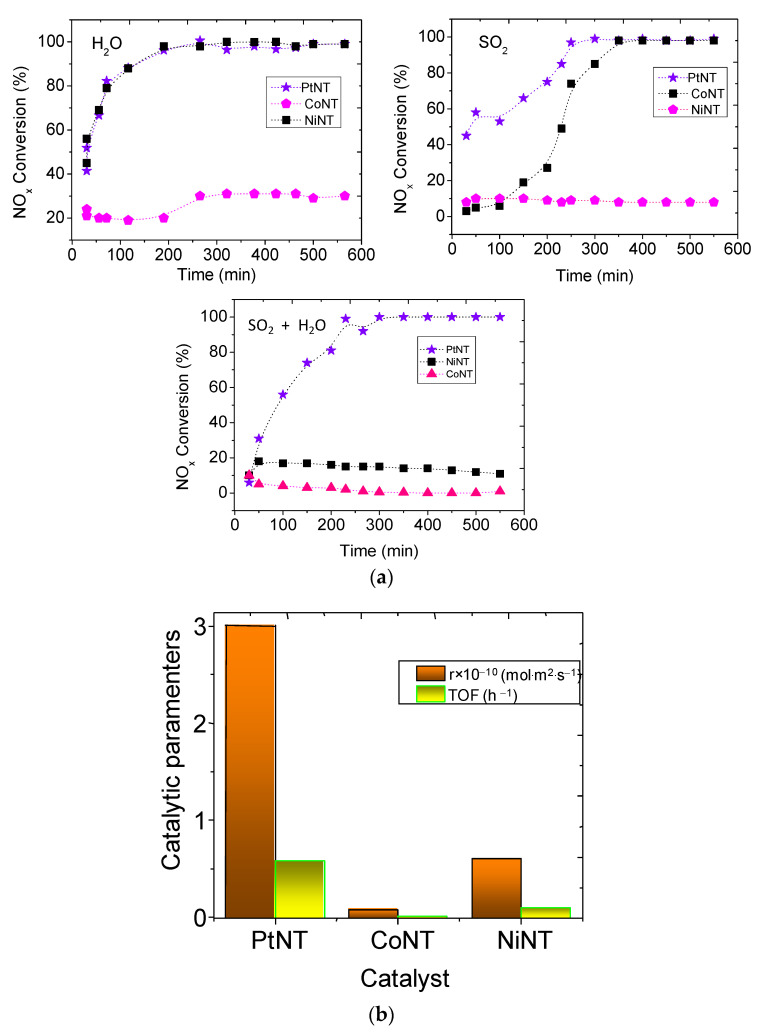
(**a**) Effects of the SO_2_ and water vapor poisons (or both) on the catalytic performance of the solids. Reaction conditions: amount of the catalyst, 0.15 g; reaction gas mixture, 500 ppm of NO, 1000 ppm of CO and balance in He; flow rate, 80 mL·min^−1^; space velocity, 48,000 h^−1^. Either 10 wt% (*v/v*) of water vapor or 50 ppm of SO_2_ (or both) was used during the reaction at 250 °C for 600 min. (**b**) Catalytic parameters determined in 6 h after the exposition of the solids to the SO_2_ and water vapor poisons.

To further demonstrate the physicochemical properties of the solids on the catalytic performance, the evaluation of the temperature reaction as a function of reaction time is shown in Figure 7a. The NaNT, CrNT, AgNT and AlNT catalysts give overall unsatisfactory results with low conversions in 600 min. of reaction because of their lack of structural stability. In consideration of the constraints of the catalysts, no further experiments are pursued using these MeNTs.

Therefore, the catalytic runs are carried out with the NiNT, PtNT and CoNT catalysts. The data clearly indicate that the solids have distinct behavior by using the SO_2_ and water vapor poisons (Figure 7a). At the early stages of the reaction, activity increases by increasing the reaction time using water vapor as a poison with NO_x_ conversion rising from 21% in 300 min to 30% at the end of the reaction for CoNT. Likewise, NO_x_ conversions over NiNT and PtNT are enhanced to values up to 40% in 150 min, and the value greatly increases to 100% running the reaction beyond this time in 600 min.Most of the findings state that the SCR reactions in the presence of water vapor poison are inhibited since the referred vapor can compete by the active sites of the catalysts through competitive adsorption causing a decay in the NO_x_ conversion at relatively low concentrations, e.g.,1–5% of H_2_O [56,57,58].

On the contrary, some reports show the role of water in SCR reaction the amount of water does not further retard the SCR reaction since new hydroxyl Brønsted acid site may be created in reason of the adsorption and decomposition of water on the solid surface [57]. In response to the contradiction, the current result illustrates that the expected NTs performance with NO_x_ conversion is superior to 20%, even upon steam introduction (Figure 7a). Thus, moisture interacts with the surface of the NTs and modifies the surface-active sites and the distribution of Lewis and Brønsted acid sites, as found elsewhere [59]. Meanwhile, a steep plateau in the NO_x_ conversion is seen in CoNT catalyst with prolonged reaction time due to Co sites oxidation by water vapor. Furthermore, the activity of NiNT and PtNT catalysts appears to be less affected by water vapor reaching up to 90% of conversion after 600 min of reaction. A consequence is that the structure of these trititanate catalysts is maintained after the catalytic test, as further demonstrated by spent catalyst characterizations.

PtNT and NiNT catalysts are found to be very tolerant to SO_2_ in CO-SCR at low temperatures. However, CoNT is less active than Pt and Ni, being limited to its low resistance against sulfur poisoning, as illustrated by the poisoning experiments. Thus, PtNT and NiNT have a NO_x_ conversion (by ~3.0–93%) within 300 min and upper times values give the complete conversion in the presence of SO_2_.

The abovementioned results encourage us to investigate the SCR performance of the most active solids regarding their tolerance of SO_2_ and H_2_O. When water vapor is introduced, the adsorption of H_2_O on the active metal sites may occur, and the Lewis acid sites might be converted into Brønsted acid sites via bonding of a water molecule, in line with the findings [60]. The literature reports also show that the SO_2_ might be harmful to the metal active sites in reason of the permanent and irreversible SO_2_ adsorption on the metal, besides the competitive adsorption of NO_x_, CO and SO_2_ for the active sites [15,56]. Another fact is that SO_2_ addition may form elemental sulfur, which probably could cover the active metal surface and block the pores causing damage in the catalyst structure [8,61]. Therefore, the NiNT deactivation is observed, when the catalyst is poisoned by SO_2_ (Figure 7b). Besides, it can be inferred that the adsorption ability of SO_2_ on the Lewis and Brønsted sites, i.e., formed by water, is much higher than that of NO_x_ and CO; hence SO_2_ may not occupy the Ni sites of NiNT leading to the catalyst deactivation. Contrary, CoNT and PtNT are resistant against SO_2_ deactivation, under the same conditions during the catalytic run. These results confirm that incorporation of Co on the NTs layered structure and the dispersion of Pt on the NTs surface provide active redox sites to the reaction. However, the well-dispersed Co in low interaction with the NTs support may provide unstable sites during the reaction, resulting in the facile poison by both water vapor and SO_2_ (Figure 7b) with the consequent limit catalytic performance of the CoNT catalyst.

PtNT catalyst has the best performance in CO-SCR at low temperatures, when water is introduced in 200 min and then cut off, and simultaneously, SO_2_ is introduced in the reaction whilst NiNT has low resistance against sulfur poisoning (Figure 7b). It can be understood that both NiNT and CoNT are extremely sensitive to the poisons, implying that the catalytic activity declined due to the lattice Co^2+^ ions oxidation by SO_2_ into the NTs structure and sulfur decomposition on Ni^2+^ sites, as well.

Our results show the reaction occurrence at lower temperature, most probably due to the formation of surface isocyanate species (NCO) on the decoration of the Pt nanoparticles on the NTs structure already at 150 °C. In the meantime, the PtNT catalyst undergoes the complete NO_x_ conversion above to 200 °C, due to the lower acidity and reduction of the Pt^2+^ sites compared to NiNT and CoNT. It is worth noticing that PtNT reduction occurs in two steps, 140–280 and 280–470 °C, respectively, attributed to the reduction of PtO_x_ species (TPR and XPS results).

When correlating with the catalytic behavior of the PtNT, one can observe that the lower conversion of NiNT may be a result of the sintering of the Ni nanoparticles and preferential CO decomposition [4] during the reaction. On the contrary, an enhancement of the PtNT activity is seen at 200 °C towards the poisons due to the Pt species stability into the NT structure, as further seen by spent catalysts characterizations. In comparison with the other solids, PtNT achieves the intrinsic rate and turnover frequency values of 3 × 10^−10^ mol m^−2^ s^−1^ and 0.56 h^−1^, respectively, after 6 h of exposure to the SO_2_ and water vapor poisons. This illustrates the influence of the Pt species in the NTs resulting in a suitable catalyst for CO-SCR reaction.

### 3.5. Spent Catalysts Characterizations

The catalysts are characterized again, after the reaction to evaluate the structural and morphological changes that they could undergo.

#### 3.5.1. Structural Features of the Spent Solids

**Figure 8:**
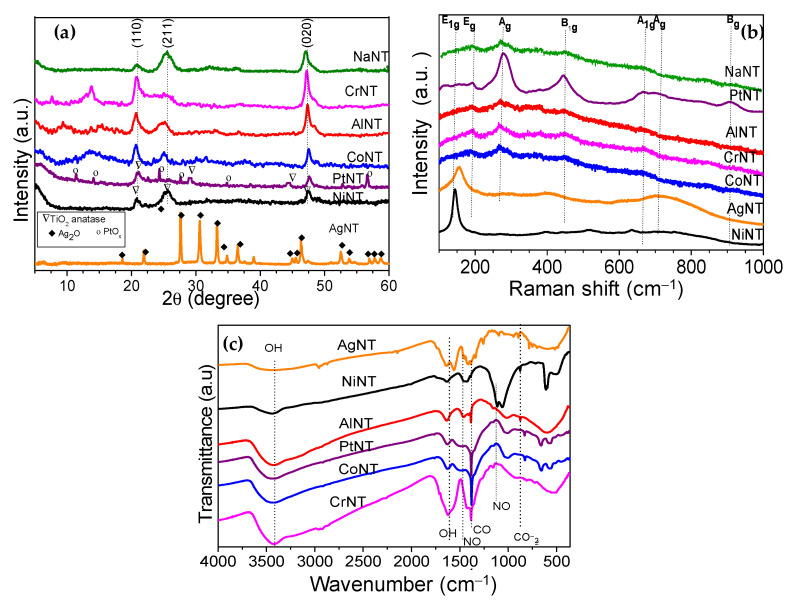
Spent characterizations of the titanate nanotubes catalysts: (**a**) XRD patterns, (**b**) Raman and (**c**) FTIR spectra. Reaction conditions: amount of the catalyst, 0.15 g; reaction gas mixture, 500 ppm of NO, 1000 ppm of CO and balance in He; flow rate, 80 mL min^−1^; space velocity, 48,000 h^−1^ at 250 °C for 600 min.

The XRD patterns of the spent catalysts tested at 250 °C after 600 min of CO-SCR reaction are depicted in Figure 8a.

Except for AgNT, all catalysts exhibit a typical NTs structure with characteristic diffraction (100), (211) and (020) planes of the monoclinic Na_2_Ti_3_O_7_ structure. As the intensity and position of the diffraction peaks of the Na_2_Ti_3_O_7_ phase in, the spent NTs have no obvious differences compared to that observed with the fresh catalysts (Figure 1a), and it can be suggested that their structure remains after the catalytic test due to the mild reaction conditions used. Some perturbations are clearly visible in the XRD peak positions as those of the (110) and (211) reflections in the CrNT, AgNT and AlNT spent catalysts, owing to the deposition of carbon species on solid surface after the catalytic test.

Additionally, the CoNT has the diffraction peaks displaced to lower 2θ regions indicating that the perturbation of the NTs structure and the formation of extra framework Co species, as well. Moreover, the peaks of the γ-AlOOH and α-CoOOH phases do not remain visible, which is all indicative of the leaching of these phases after the reaction. It is noted that the intensity of the characteristic peaks of the cubic Ag_2_O is present in the stable AgNT structure. In the case of the NiNT, peaks from the NTs phase along with TiO_2_ anatase are seen. Moreover, there is no Ni species visible since the intercalation of these species in the NTs interlayer region remains. Moreover, the TiO_2_ anatase phase is produced by the partial calcination of the solid, as a result of both intercalated and surface Ni^2+^ species with Ti^3+^ interactions under the reaction conditions evaluated. For spent PtNT, certain features of the trititanate and TiO_2_ anatase phases are seen in the XRD pattern besides some narrow peaks associated with the PtO_x_ species. This suggests that a high fraction of Pt^2+^ species is exposed after the catalytic test.

Regarding the NO_x_ conversions being in the sequence of PtNT > NiNT > CoNT > CrAlNT ~ AlNT > NaNT at 250 °C (Figure 6), it suggested that the metals such as Ni, Pt and Co are active components remains in the NTs, which results in the maintenance of their structure. Conversely, Cr, Ag, Na and AlNT have poor catalytic performance, and this may be ascribed to lesser accessibility of these species to the reactants at 250 °C, despite their structure being retained after the reaction.

The Raman spectra of metal catalysts are examined after the CO-SCR reaction. Figure 8b depicts the spectra recorded after using the samples for 1 h at 250 °C. The NaNT, AlNT, CoNT, PtNT and CrNT spectra exhibit broad prominent bands at around 236, 353, 500, 514 (shoulder), 669 (shoulder), 681, 815, 850 and 958 cm^−1^. By comparison with Figure 1b, some of these bands are closely related to the 276 (Ag), 445 (B1g), 656 (A1g), 696 (Ag) and 908 (Bg) cm^−1^ vibrational modes of the trititanate structure, in line with the literature reports [4,25]. The similarity of the NiNT and AgNT spectra indicates that trititanate structures would be coexisting with the TiO_2_ anatase ones with the main Raman modes at 144, 402 and 650 cm^−1^, in line with the findings [62]. Specifically, for AgNT, the Ag–O vibration from Ag_2_O phase at around 561, 700 and 1010 cm^−1^ still detected [32]. In the case of NiNT, the bands are broader and more intense than those of AgNT, and thus, the modes ascribed to be from TiO_2_ anatase stretching are more evident. These results confirm the XRD results that demonstrate that the structures of the NaNT, AlNT, CoNT, PtNT and CrNT solids are not affected after the catalytic test and ascertain the catalytic performance of these solids due to their stability due to the mild conditions used during the reaction. However, the vibrational modes of the trititanate structure are vanished from the AgNT and NiNT either causing the phase transformations or forming organic other adsorbed compounds. This is further confirmed by FTIR and SEM-EDS analyses.

FTIR spectra of the solids are also examined after the reaction (Figure 8b). All spent solids are dominated by the significant absorptions of OH vibrations at about 3600 and 1640 cm^−1^ from the stretching and bending vibrations of the OH groups, respectively. This assignment may be due to the physically adsorbed water and the structural OH groups [13]. Several bands are seen in the 3000−1000 cm^−1^ region in comparison with the spectra of the fresh solids (Figure 1c).

The weak bands at around 1585 cm^−1^ arises as νNO from bidentate nitrates, which results in the NO_x_ decomposition [6,13]. The bands at 1060, 1347 and 1557cm^−1^ are ascribable to the carbonates *ν_as_*(CO_2_^−^) from CO dissociation onto the surface as a consequence of the methanation or Water Gas Shift (WGS) reactions [4,6]. The low intensity of these bands evidences a small amount of carbonates adsorbed on solid surface. The formation of carbon species deposits on AgNT, CrNT and AlNT spent catalysts is likely. Such reactions are possibly related to the deactivation behavior of these solids. Additionally, the nitrite adsorbed species arising from the oxidation of NO on PtNT and NiNT sites to NO_2_ are visible at 1292 and 1255 cm^−1^ (ν_asym_NO_2_) and concomitantly with the 1030–1000 cm^−1^ range (ν_sym_NO_2_), as well. The bands associated with the (ν_asym_NO_3_) are depicted at 1320 and 1420–1000 cm^−1^ from nitrates formed during the reaction [63]. These absorption bands are observable over NiNT and PtNT samples, indicating the preferential adsorption of NO_x_ on these solids. As found elsewhere, the nitrates species are seen at around 1418, 1307, 1020 cm^−1^ whereas the chelates are visible at around 1552 cm^−1^ when adsorbing NO_x_ at around 350 °C on Pt-based samples [6,63].

#### 3.5.2. Morphological Features of the Spent Solids

**Figure 9:**
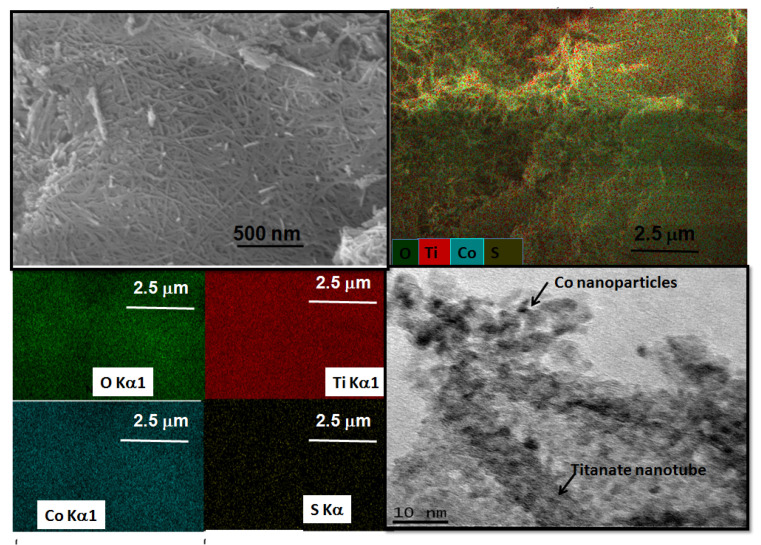
SEM-EDS and TEM images of CoNT spent titanate nanotubes catalyst characterizations. Reaction conditions: amount of the catalyst, 0.15 g; reaction gas mixture, 500 ppm of NO, 1000 ppm of CO and balance in He; flow rate, 80 mL·min^−1^; space velocity, 48,000 h^−1^. Either 10 wt% (*v/v*) of water vapor or 50 ppm of SO_2_ (or both) was used during the reaction at 250 °C for 6 h.

**Figure 10:**
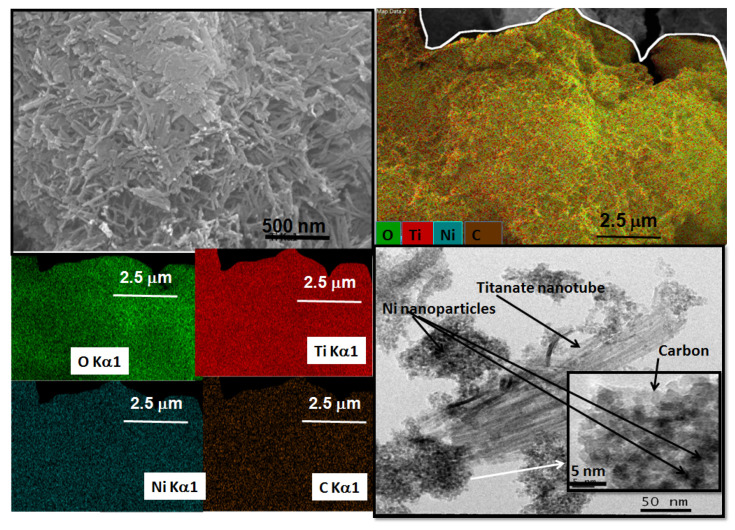
SEM-EDS and TEM images of NiNT spent titanate nanotubes catalyst characterizations. Reaction conditions: amount of the catalyst, 0.15 g; reaction gas mixture, 500 ppm of NO, 1000 ppm of CO and balance in He; flow rate, 80 mL·min^−1^; space velocity, 48,000 h^−1^. Either 10 wt% (*v/v*) of water vapor or 50 ppm of SO_2_ (or both) was used during the reaction at 250 °C for 6 h.

**Figure 11:**
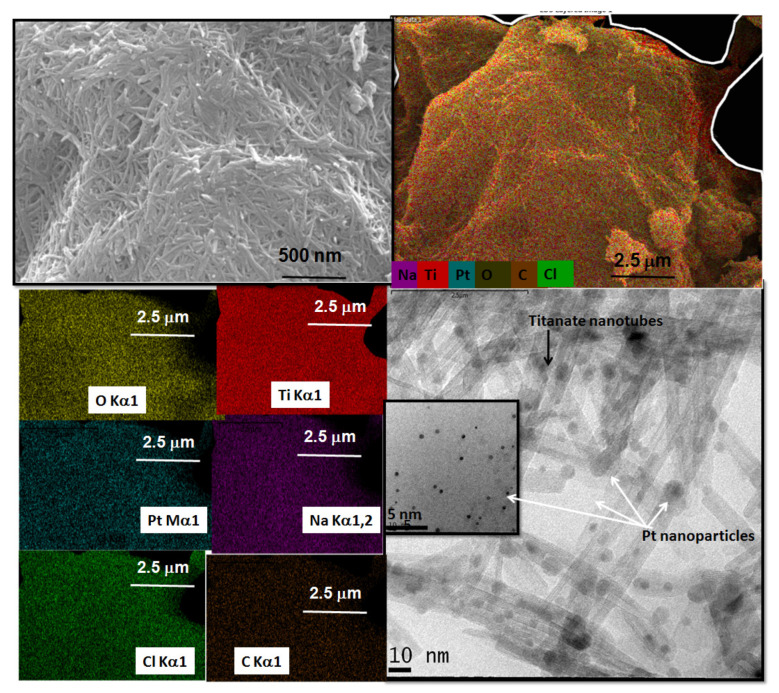
SEM-EDS and TEM images of PtNT spent titanate nanotubes catalyst characterizations. Reaction conditions: amount of the catalyst, 0.15 g; reaction gas mixture, 500 ppm of NO, 1000 ppm of CO and balance in He; flow rate, 80 mL·min^−1^; space velocity, 48,000 h^−1^. Either 10 wt% (*v/v*) of water vapor or 50 ppm of SO_2_ (or both) was used during the reaction at 250 °C for 6 h.

SEM-EDS images confirm observations showing the morphological evolution by poisoning of the CoNT, NiNT and PtNT solids. The morphologies of the spent solids (Figure 9, Figure 10 and Figure 11) are distinct from those of fresh catalysts (Figure 2b and Figure 3f,g). Accordingly, the fresh CoNT exhibits a slight aggregation of the nanotubes forming a rough surface [4], before the reaction. Instead, more aggregated titanates nanotubes particles morphology is clearly visible when CoNT is used at 250 °C for 600 min. in the presence of SO_2_ and water vapor poisons, as shown in Figure 9 through SEM image. Moreover, EDS mapping illustrates a well dispersion of the oxygen, titanium and cobalt, elements of ca. 55.6, 31.7, 0.3%, respectively. Moreover, an enormous amount of sulfur deposits of ca. 12.4% is seen on solid surface. As evidenced by the appearance of a significant amount of sulfur and no carbon, the cobalt species are easily oxidized in the reaction media. The sulfur deposits on surface favor the inaccessibility to the Co active sites, which is reasonably responsible for the catalytic activity in CO-SCR. Moreover, the sulfur deposition does not significantly affect the tubular structure, and one can see the Co nanoparticles having sizes in the 1–5 nm range intercalated (decorated) on the nanotubes, as illustrated by TEM image. Hence, low SO_2_ tolerance is the leading cause of the deactivation of the CoNT catalyst. The cobalt is well known as very smooth oxidant for CO involved reactions [4], and thus, the oxidation of the Co^2+^ species along with the sulfur formed during the experiments impedes either NO_x_ or CO adsorption on the active sites resulting in the deactivation of the solid.

In the case of spent NiNT (Figure 10), unlike CoNT, on which NO_x_ conversion is decreased during the poisoning experiments, SEM images illustrate that the titanate nanotubes morphology remains with the tubes entangled forming a dense plate, in line with Raman and XRD results. For further ensuring the uniform dispersion of each element in the spent catalysts, the EDS elemental mapping images indicate that each element (e.g., Na, Ti and O) is uniformly distributed throughout the solid surface. Moreover, Ni particles are clearly visible on solid surface with C coming from the CO decomposition parallel reactions on metallic Ni nanoparticles during the reaction [4]. Moreover, the sulfur observed by EDS is 0.0% on NiNT surface. Previous studies have demonstrated the Ni ability of forming coke under relatively mild condition, when CO is present in catalytic reactions [4,63]. Noteworthy, TEM image depicts the tubular morphology of the titanate nanotubes along with a bundle of Ni nanoparticles. Indeed, these nanoparticles of about 1–10 nm remain strongly adhered on the surface of the tubes possessing uniform diameters of around 4–50 nm. Moreover, carbon species around the Ni nanoparticles are observable throught the HRTEM images, which could be indicative of the presence of generous carbon deposition, which accords to FTIR results. Thus, the performance of the NiNT catalyst in the SCR-CO reaction (Figure 6) is due to the Ni nanoparticles, but the modest performance is mainly due to the carbon deposits on solid surface and sintering of Ni particles, as well.

To further investigate the stability of PtNT towards the CO-SCR reaction and correlate its structural and morphological features to justify its better performance in the reaction, SEM-EDS and TEM images are performed after the poisoning experiments. The SEM micrograph of spent PtNT (Figure 11) suggests that the morphology of the sample is not altered, after the reaction with the appearance of the entangled nanotubes. Through EDS analyses, Pt nanoparticles as PtO_x_ and Pt-chlorinated species are homogeneously dispersed on the NT surface. Moreover, traces of Na appear as a result of the remaining Na_2_Ti_3_O_7_ phase concomitantly with anatase TiO_2_ presence while Cl comes from the chlorinated Pt species. The HRTEM micrograph somewhat evidences the tubular morphology maintenance with the dark dots corresponding to strikingly dispersion Pt nanoparticles on the external surface of the tubes. These particles are indeed smaller, e.g., 1–5 nm (Figure 11 included) than those NiNT, as expected for Pt-supported based samples.

## 4. Conclusions

Titanate nanotubes containing metals were prepared with the assistance of the ion exchange and impregnation procedures. The structural and compositional characterizations through Raman, FTIR spectroscopy, XRD and HRTEM and SEM-EDS analyses revealed that the sodium from Na_2_Ti_3_O_7_ structure was replaced by the Co, Pt, Ni, Al and Cr ions in the NTs interlayer regions. Contrary, Ag ions were entirely deposited on NTs surface forming Ag_2_O phase. Besides, the oxy hydroxides of Al, Ni, Co and Cr were observed as extra framework phases along with Pt oxyhydroxides and Pt-chlorinated species.

The Ni, Co and Pt species had important implications on the catalytic performance of the solids by improving the NO_x_ conversion, but Al, Cr and Ag species contributed to their modest performance in the NO_x_ conversion through CO-SCR reaction at distinct temperatures. The catalytic activity of CoNT, NiNT and PtNT catalysts was more stable in the CO-SCR reaction towards the water vapor and SO_2_ poisons with the former catalyst having moderate conversions. The Co species in CoNT nanotubes showed low NO_x_ conversions during the poisoning runs; most of the exposed Co nanoparticles catalyze the oxidation reactions, and sulfur deposition on CoNT surface provoked its deactivation. The presence of severe carbonaceous deposition on the NiNT revealed the low stability of the catalyst. On the other hand, intimate synergism between the Pt nanoparticles and the titanate nanotubes structured were considered to be one of the reasons for the superiority of PtNT and in the CO-SCR reaction towards SO_2_ and water vapor poisons.

## Figures and Tables

**Table 1:** Physicochemical properties of the catalysts studied. The Na/Ti molar ratios are obtained by EDS analyses whereas the interwall distances are taken through XRD measurements.

Catalysts	Na/Ti ^a^ Molar Ratio	*d* ^b^ (nm)
NaNT	0.57	0.84
CoNT	0.02	0.91
CrNT	0.03	0.89
AlNT	0.06	0.90
NiNT	0.03	0.89
PtNT	0.03	0.90
AgNT	-	-

^a^ Obtained by EDS analyses; ^b^ Interwall distance determined by XRD.

**Table 2:** Textural properties determined from the nitrogen physisorption isotherms for the catalysts studied.

Catalyst	BET Surface Area (m^2^·g^−1^)	Pore Volume (cm^3^·g^−1^)	Pore Diameter (nm)
NaNT	189	0.62	15
CoNT	468	0.77	5.9
AlNT	482	0.89	6.3
CrNT	293	0.65	7.8
PtNT	180	0.59	5.9
NiNT	217	0.71	6.3
AgNT	206	0.18	4.3

**Table 3:** Binding Energies (in eV) of the C 1*s*, Ti 2*p*_3/2_, O 1*s*, Na 1*s*, Co 2*p*_3/2_, Pt 4*f*_7/2_ and Ni 2*p*_3/2_ signal for the studied catalysts.

Catalysts	C 1*s*	O 1*s*	Ti 2*p*_3/2_	Na 1*s*	Co 2*p*_3/2_	Ni 2*p*_3/2_	Pt 4*f*_7/2_
CoNT	284.8 (76) 286.6 (18) 289.1 (6)	530.3 (91) 532.2 (9)	458.7	1071.4	781.0	-	-
NiNT	284.8 (73) 286.6 (18) 289.1 (9)	530.3 (91) 532.1 (9)	458.6	1071.6	-	855.9	-
PtNT	284.8 (70) 286.6 (12) 289.1 (18)	530.3 (91) 531.9 (9)	458.7	1072.0	-	-	72.5 (88) 75.3 (12)

**Table 4:** General features of the solids investigated by TPR and acidity measurements of the CoNT, NiNT and PtNT fresh solids.

Catalyst	Temperature of Reducibility by H_2_ TPR (°C)	Temperature of CO_2_ Desorption by CO_2_ TPD (°C)	Acidity by Pyridine-TPD (μmol Py·gcat^−1^)
NiNT	400–600	100–300; >300	103
PtNT	200; >700	100–250; >300–500	261
CoNT	150; >350	100–300; >300	93

## Data Availability

The data presented in this study are available on request from the corresponding author.
