# Correction: Carmo et al. Effects of the Incorporation of Distinct Cations in Titanate Nanotubes on the Catalytic Activity in NO_x_ Conversion. *Materials* 2021, *14*, 2181

**DOI:** 10.3390/ma15041423

**Published:** 2022-02-15

**Authors:** José Vitor C. do Carmo, Cleanio L. Lima, Gabriela Mota, Ariane M. S. Santos, Ludyane N. Costa, Anupama Ghosh, Bartolomeu C. Viana, Monique Silva, João M. Soares, Samuel Tehuacanero-Cuapa, Rossano Lang, Alcineia C. Oliveira, Enrique Rodríguez-Castellón, Elena Rodríguez-Aguado

**Affiliations:** 1 Department of Analytical and Chemical-Physic Chemistry, Pici Campus-Block 940, Federal University of Ceará, Fortaleza 60040-531, Brazil; vitor.costa@alu.ufc.br (J.V.C.d.C.); gabrielamotab@alu.ufc.br (G.M.); 2 Material Science and Engineering & Physics Department, Federal University of Piauí, Teresina 64049-550, Brazil; cleanio@ufpi.edu.br (C.L.L.); arianeo_1q@bol.com.br (A.M.S.S.); luydiane@bol.com.br (L.N.C.); anupama1984@gmail.com (A.G.); bartolomeu@ufpi.edu.br (B.C.V.); 3 Fortaleza Campus, Federal Institute of Education—IFCE, Av. 13 de Maio, 2081, Benfica, Fortaleza 60040-531, Brazil; moniquessouza22@gmail.com; 4 Physics Department, State University of Rio Grande do Norte-UERN, BR 110-km 48, R. Prof. Antônio Campos, Costa e Silva, Mossoró 59610-210, Brazil; joaomsoares@gmail.com; 5 Central Microscopy Laboratory, Physics Institute—UNAM, Research Circuit s/n, University City, Coyoacán, Mexico City 04510, Mexico; samueltc@fisica.unam.mx; 6 Institute of Science and Technology—ICT, Federal University of São Paulo—UNIFESP, São José dos Campos 12231-280, Brazil; rossano.lang@unifesp.br; 7 Department of Inorganic Chemistry, Faculty of Science, University of Málaga, 29071 Málaga, Spain; aguadoelena5@gmail.com

In the original publication, there was a mistake in Figure 1 as published. Figure 1 is in duplicity with Figure 8 in the paper. The corrected Figure 1 appears below. The authors apologize for any inconvenience caused and state that the scientific conclusions are unaffected. The change does not affect the interpretations of the data already included in the published paper. The original publication has also been updated [1].

## Figures and Tables

**Figure 1:**
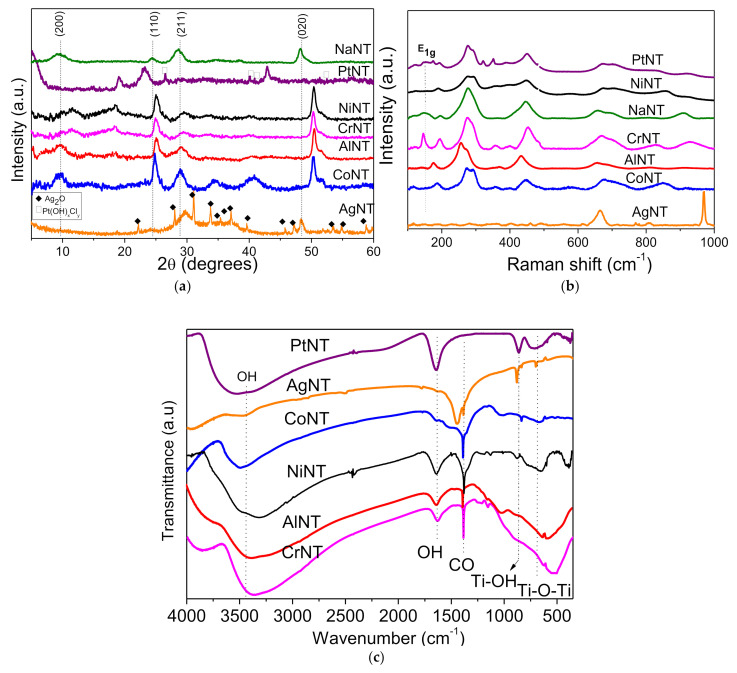
(**a**) XRD diffractograms, (**b**) Raman and (**c**) FTIR spectra of the catalysts studied. The main hkl reflections of Na_2_Ti_3_O_7_ are shown in parenthesis above the diffraction lines.
